# Defensive caesarean section: A reality and a recommended health care improvement for Romanian obstetrics

**DOI:** 10.1111/jep.13025

**Published:** 2018-09-04

**Authors:** Cringu Antoniu Ionescu, Mihai Dimitriu, Elena Poenaru, Mihai Bănacu, Gheorghe Otto Furău, Dan Navolan, Liana Ples

**Affiliations:** ^1^ Department of Obstetrics and Gynecology “St Pantelimon” Clinical Emergency Hospital ‘Carol Davila’ University of Medicine and Pharmacy Bucharest Romania; ^2^ Department of Obstetrics and Gynecology “St Pantelimon” Clinical Emergency Hospital ‘Carol Davila’ University of Medicine and Pharmacy Bucharest Romania; ^3^ Complementary Sciences, Medical Informatics and Biostatistics Discipline “Carol Davila” University of Medicine and Pharmacy Bucharest Romania; ^4^ Department of Obstetrics and GynecologyGynaecology “St Pantelimon” Clinical Emergency Hospital ‘Carol Davila’ University of Medicine and Pharmacy Bucharest Romania; ^5^ Medicine Faculty, Department of Obstetrics and Gynecology, Emergency District Hospital West University Arad Romania; ^6^ Department of Obstetrics and Gynecology, City Emergency Clinical Hospital Victor Babes University of Medicine and Pharmacy Timisoara Romania; ^7^ Department of Obstetrics and Gynecology “St Ioan” Clinical Emergency Hospital “Carol Davila” University of Medicine and Pharmacy Bucharest Romania

**Keywords:** caesarean delivery on maternal request, defensive caesarean section, defensive medicine, malpractice

## Abstract

**Rationale:**

Defensive caesarean section (CS) has become one of the most common medical procedure worldwide. Additionally, performing CS in accordance with the patient's choice is an appropriate professional practice.

**Aims and Objective:**

This paper reports a prospective, observational, multicenter study to quantify the use of this type of practice that is performed by obstetricians to avoid medico‐legal complaints and decrease the frequency of malpractice litigations.

**Methods:**

We interviewed 73 obstetricians from three distinct units of obstetrics and gynaecology, to assess their opinion regarding defensive caesarean delivery and caesarean delivery performed upon maternal request. We conducted an opinion‐based survey using questionnaires based on nine, close‐ended questions.

**Results:**

Out of 73 respondents, 51 (69.9%) stated that they perform defensive CS; 63 (86.3%) declared that their choice of birth delivery is influenced by the risk of being accused of malpractice; 60 (82.2%) indicated that it is normal for the patient to be able to decide on the type of delivery; and 63 (86.3%) declared that they consult their patients regarding their delivery preferences. We found statistically significant differences between the respondents who declare that they perform defensive CS (69.9%) and those who said that they are influenced by the risk of malpractice when they choose the method of delivery for their patients (86.3%) (*P* < .001; McNemar Test).

**Conclusions:**

The results of our study indicate that defensive caesarean section is a widespread practice among obstetrics practitioners in Romania.

## 1 INTRODUCTION

A natural and predictable outcome of free market medicine is the practice of defensive medicine. In obstetrics, this is most often seen as “the defensive cesarean section (CS)”.^1^

In this study, the idiom “defensive CS” (or CS with a defensive indication) is defined as a caesarean delivery recommended by the doctor in the absence of any clear medical indication that such a delivery method is needed to avoid possible litigation or a possible accusation of malpractice.^1^ This definition was proposed because no other clear definition currently exists. Some authors have defined defensive CS as a caesarean section performed by the doctor to avoid a lawsuit rather than for the benefit of the patient, with such a practice being considered both legitimate by some and immoral by others.^2^ There is a difference between defensive CS and CS on maternal request. Caesarean delivery on maternal request is defined as a primary caesarean delivery performed at the request of the mother in the absence of any medical or obstetric indications.^2^, ^3^ There are insufficient data to evaluate the benefits and risks of CS on maternal request compared to vaginal delivery and more research is needed. Therefore, any decision to perform a CS on maternal request should be carefully analysed, individualized, and be consistent with ethical principles.^4^ Recently, the support for the physician's decision to implement an informed pregnant patient's request for caesarean delivery in the absence of an accepted medical indication has been increasing.^5^, ^6^, ^7^ Therefore, it is now considered ethically acceptable to perform CS in a well‐informed patient who has provided consent, and this is thus considered good professional practice.

Defensive CS delivery can be considered an example of defensive medicine, which is defined as the deviation of the medical behavior from medical protocols or guidelines to reduce the number of complaints or criticisms from the patients.^3^ Summerton described this approach to general medical practice as the ordering of tests, treatments and procedures to protect the doctor from criticism rather than correctly diagnosing and treating the patient.^3^ Defensive medical practice can be either positive or negative. The distress produced by fear of possible litigation results in the doctor declining to treat particular patients or to perform certain risky procedures. However, using tests and initial treatments inherent to defensive medicine makes it possible to reduce the risk of injury that leads to malpractice complaints.^4^

Obstetrics and surgery are perceived as the specialties that are most vulnerable to malpractice claims.^4^ In obstetrics, the risk of malpractice litigation increases because there are at least two patients: the pregnant woman (mostly young and healthy) and her newborn or newborns (socially perceived and accepted as a distinct patients). Additionally, pregnancy, parturition, and subsequent hospitalization are considered by the general population to be routine medical procedures that are associated with a positive outcome. Accordingly, pregnancy and delivery are generally not perceived as potentially dangerous, although there are risks with vaginal delivery as well as with CS.

Therefore, the fear of a malpractice lawsuit would increase the incidence of CS.^5^ In some studies, it was estimated that among deliveries, 27.5% were performed as CS, of which 6.6% were performed because of legal considerations, not because of strict medical indications.^6^ Other studies have assessed the gradual increase in the incidence of elective CS in Western Europe along with the increase in defensive medical practices by obstetricians. They concluded that defensive medicine in obstetrics is deeply rooted in the everyday practices of obstetricians and gynaecology physicians.^7^ Additionally, the morbidity associated with vaginal delivery is considered socially unacceptable, because the general perception is that CS delivery is safer.^8^ Patients' lack of information on CS often gives them the perception that this type of delivery is only associated with benefits. In 2014, Romania had the third highest CS rate (38%) in Europe, 13% less than that of Turkey which had a CS rate of 50.36%, and close to Italy, which had a CS rate of 38.81%.^9^

In the current study we report a multicenter prospective study that measured the number of defensive CS performed in three tertiary medical units in Romania. The secondary objective was to test whether introducing caesarean delivery upon maternal request would reduce the incidence, at least in part, of such litigations, and to investigate the defensive practices that result from the potential threat of litigation.

## 2 METHODS

### 2.1 Study design

We prospectively analysed the opinions of practicing obstetricians from three distinct university obstetrics and gynaecology departments of tertiary referral centers in Romania that treat the most complex obstetrical cases: St. Pantelimon Clinical Emergency Hospital of Bucharest, Bucur Maternity Hospital of Bucharest St. Ioan Clinical Emergency Hospital, and Vasile Goldiş Western University of Arad, Clinical University Hospital. Two of the hospitals are in different geographical regions in the capital city, and the third one is in the northern part of the country. The total number of practicing obstetricians with a licence to practice in the three hospitals was 73.

Our observational, prospective cohort study gathered the opinions of 73 active obstetricians (*N* = 73) through a voluntary anonymous opinion‐based questionnaire that was composed of nine questions. The interview session occurred simultaneously during their morning meetings in all the three centers during the period 1 September 2016 to 1 October 2016. The study was approved by the Ethics Committees of each of the tertiary centers, and informed consent was obtained from the participants after they had completely understood the intended use of the data they provided.

### 2.2 Data collection

The inclusion criteria were as follows: practicing obstetrician with a licence to practice in Romania, affiliation with one of the three departments where the study was conducted and freely and voluntarily agreeing (unremunerated and unrewarded materially or otherwise) to participate in the study and to provide truthful information (anonymously) with results that would be included in scientific works. The only exclusion criterion was the freely expressed refusal of the interviewed obstetrician to participate in the study. Visiting obstetricians were excluded from the survey.

Voluntary completion of the opinion‐based questionnaire represented written informed consent to participate in the study and agreement to the publication of its results. The inclusion and exclusion criteria did not discriminate based on ethnicity, nationality, professional status, age, sex, socio‐cultural background, social status, religion, political beliefs, race, or sexual orientation. Furthermore, no clinicians refused to participate in the study. Participation in the study was entirely voluntary. To guarantee anonymity, which contributed, in our view, to the honesty of the answers, we did not collect or process any data that could lead to the identification of the respondents. The questionnaire consisted of nine questions not included in the obstetrics literature. The independent variables were sex, age, and professional data (specialists and residents). The dependent variable was the performance of defensive CS, the extent of defensiveness among obstetricians, and the physician‐patient relationships influenced by the concern for legal demands. We used “yes” or “no” answers in the close‐ended questionnaire to enhance its clarity because there were no multiple variables and the doctors had experienced only one of the options. The actual number of participants is representative of the obstetricians in Romania because their age varied from 24 to over 66 years, their obstetric expertise varied between 0 and 36 years, and the number of women was 43 and number of men was 30.

The first three questions of the questionnaire were related to the respondent's sex, age, and years of practicing obstetrics (age group and seniority). Question 4 asked the respondent whether it is normal (to be answered as “yes” or “no”) for the patient to be able to choose the type of delivery. Question 5 asked the obstetrician if he/she agreed with the legalization of CS delivery on maternal request (to be answered as “yes” or “no”). Question 6 asked the respondent whether he/she asks patients about their preference regarding delivery (to be answered as “yes” or “no”). Question 7 was “Have you ever performed a CS for defensive purposes, only? (to avoid a possible malpractice lawsuit against you)?” (to be answered as “yes” or “no”). Question 8 asked the respondent to self‐identify the number of defensive CS deliveries performed as a percentage of the total number of CS deliveries performed (0%, 1‐9%, 10‐20%, 21‐50%, and over 50%). Question 9 asked the obstetrician “When choosing the method of birth for the patient, do you consider that you are influenced in this choice by the risk of being accused of malpractice?” (to be answered as “yes” or “no”). The difference between question 7 and 9 was that in question 9 the main objective was to investigate if the risk of a malpractice lawsuit influenced the choice of the obstetrician.

### 2.3 Statistical analysis

The answers of the 73 interviewed obstetricians were analysed using the SPSS software version 23 and EpiInfo 3.5.4. The data analysed were synthesized as frequencies as well as percentages. For the binary categorical data, the Fisher Exact Test and Pearson chi‐square Test were used (when 20% of the expected frequencies were less than 5), and for the non‐binary categorical data, the Likelihood Ratio Test was used. The McNemar Test was used to compare binary pair data. The significance level was set to 5%.

## 3 RESULTS

The structure of the respondent obstetrician group was as follows: 35/73 (47.9%) respondents from Arad Clinical University Hospital, 18/73 (24.7%) from Bucur Maternity (Bucharest), and 20/73 (27.4%) from St. Pantelimon Hospital (Bucharest). Of the respondents 43 were women (58.9%) and 30 men (41.1%). The ages of the respondents were as follows: those with age 24 to 30 years = 17/73 (23.3%); age 31 to 35 years = 15/73 (20.5%); age 36 to 40 years = 7/73(9.6%) age 41 to 50 years = 14/73(19.2%); age 51 to 60 years = 11/73(15.1%); age 61 to 65 years = 6/73(8.2%); age 66 years or over = 3/73 (4.1%). The obstetrics expertise of the respondents were as follows: those with experience of 0 to 5 years (resident doctors still in the process of training but with a free practice licence under guidance) = 28/73 (38.4%); 6 to 10 years (young specialists and head physicians) = 8/73 (11.0%); 11 to 20 years (obstetricians with full professional recognition) = 14/73 (19.2%); 21 to 30 years (obstetricians with considerable experience) = 15/73 (20.5%) 31 to 35 years (obstetricians at the end of their career and with substantial experience) = 2/73 (2.7%); and 36 years or over (obstetricians close to their retirement or with an extended practice licence after the retirement) = 6/73 (8.2%).

Sixty of the 73 respondents (82.2%) considered it to be normal for the patient to decide on the type of delivery (the other 13/73 responded negatively). Fifty‐seven of the 73 (78.1%) agreed with the legalization of CS delivery upon maternal request (the other 16/73 expressed their disagreement). Of the 73 obstetricians, 63 (86.3%) declared that they ask their patients about their preferred method of delivery (the other 10/73 said they do not ask, so they are not influenced).

Fifty‐one out of the 73 obstetricians (69.9%) declared that they perform defensive caesarean delivery (the other 22/73 said they do not perform this delivery method), and 63/73 (86.3%) said they are influenced in choosing the delivery method by the risk of being accused of malpractice (the other 10/73 declared they are not influenced).

Of the 51 of the 71 obstetricians (69.9%) who declared that they perform defensive CS, 23 (45.09%) said that this type of delivery represented 10% to 20% of the total number of the CS deliveries performed, 10 (19.6%) declared that more than 50% of the CS deliveries performed were defensive, and nine respondents (17.64%) indicated that 1% to 9% or 21% to 50% of the total number of caesareans they performed were defensive CS deliveries. Only 22/73 of the respondents (30.1%) said they do not perform defensive CS.

In the answers given by the respondents from the centers used in the study, we detected two statistically significant differences: the percentage of doctors at Arad and Bucur who stated that they ask their patients the preferences that they have regarding delivery (32/35 = 91.4% in Arad and 11/18 = 61.2% in Bucur: 0.001014 likelihood ratio) and the percentage of physicians from Arad and St. Pantelimon who stated that they perform defensive caesarean deliveries (20/35 = 57.1% at Arad and 19/20 = 95.0% at St. Pantelimon; Pearson Chi‐Square 0.012405).

By comparing the responses given by the respondents, we did not detect any statistically significant difference for questions 4 to 9 of the questionnaire in terms of the gender of the respondent; however, women were more likely to agree to caesarean legalization on maternal request (women: 36/43 = 83.7% versus male: 21/30 = 70.0%; Pearson Chi‐Square 0.163240). We also found no statistically significant difference when comparing the answers to questions 4 to 9 with reference to the age of the respondents.

Variations in the answers to the six questions of the questionnaire (questions 4‐9) with respect to the experience in practicing obstetrics are shown in Table 1; we recorded statistically significant differences regarding the affirmative answer to question 7 (performing the defensive CS) given by those with 0 to 5 years and 11 to 20 years of experience (12/28 = 42.9% who performed CS deliveries vs 13/14 = 92.9% who performed CS deliveries; likelihood ratio 0.000775).

**Table 1:** Variation of the response to questions 4 to 9 according to the number of years of experience in practicing obstetrics

Seniority in Obstetrics (y)	0‐5 Years (*N*)/%	6‐10 Years *N*/%	11‐20 Years *N*/%	21‐30 Years *N*/%	31‐35 Years *N*/%	36 Years or over *N*/%	*P*‐Value
You feel that it is normal for the patient to be able to choose the type of delivery = Yes	24/28 (85.7%)	6/8 (75.0%)	12/14 (85.7%)	12/15 (80.0%)	1/2 (50.0%)	5/6 (83.3%)	0.88 likelihood ratio
You agree with caesarean legalization on maternal request = Yes	22/28 (78.6%)	7/8 (87.5%)	10/14 (71.4%)	12/15 (80.0%)	1/2 (50%)	5/6 (83.3%)	0.89 likelihood ratio
You ask the patients how they prefer to deliver = Yes	22/28 (78.6%)	7/8 (87.5%)	13/14 (92.9%)	14/15 (93.3%)	2/2 (100%)	5/6 (83.3%)	0.66 likelihood ratio
You perform defensive caesarian practice = Yes	12/28 (42.9%)	8/8 (100%)	13/14 (92.9%)	11/15 (73.3%)	2/2 (100%)	5/6 (83.3%)	<0.01 likelihood ratio
Percentage of defensive caesarean among the total number of personal caesareans							0.09 likelihood ratio
0%	16/28 (57.1%)	0/8 (0.0%)	1/14 (7.1%)	4/15 (26.7%)	0/2 (0.0%)	1/6 (16.7%)	
1%‐9%	2/28 (7.1%)	1/8 (12.5%)	3/14 (21.4%)	3/15 (20.0%)	0/2 (0.0%)	0/6 (0.0%)	
10%‐20%	7/28 (25.0%)	3/8 (37.5%)	6/14 (42.9%)	4/15 (26.7%)	1/2 (50.0%)	2/6 (33.3%)	
21%‐50%	1/28 (3.6%)	2/8 (25.0%)	2/14 (14.3%)	2/15 (13.3%)	0/2 (0.0%)	2/6 (33.3%)	
Over 50%	2/28 (7.1%)	2/8 (25.0%)	2/14 (14.3%)	2/15 (13.3%)	1/2 (50.0%)	1/6 (16.7%)	
Are you influenced in the choice of choosing the delivery method by the risk of being accused of malpractice = Yes	21/28 (75.0%)	8/8 (100%)	13/14 (92.9%)	14/15 (93.3%)	2/2 (100%)	5/6 (83.3%)	0.23 likelihood ratio

Notes: *P*‐value, significance level was set for *P* < .05.

Abbreviations: y, years; N, number of obstetricians (respondents); %, percentage of obstetricians (respondents).

When comparing the answers to questions 4 and 5, we did not find statistically significant differences between the answers of those who consider it normal for the patient to choose the type of delivery (82.2%) and those who agree with the legalization of CS on maternal request (78.1%) (*P*‐value = 0.51 McNemar Test); however, we found statistically significant differences between the percentage of those agreeing with caesarean section legalization on maternal request for the group who answered that it is normal for the patient to choose the method of delivery (90.0%) and for the group who did not consider it normal for the patient to choose the method of delivery (23.1%) (*P*‐value < .001 Fischer Exact Test).

By comparing the answers given to questions 4 and 6, we found no statistically significant differences between the answers of those who considered it normal for the patient to choose the method of delivery (82.2%) and the answers of those who asked their patients how they want to deliver (86.3%) (p‐value = 0.51 McNemar Test); however, we found statistically significant differences among those who asked their patients how they want to deliver, and those who considered it normal for the patient to be able to decide how to deliver (95.0%) and those who did not consider it normal for the patient to be able to choose the mode of delivery (46.2%) (*P*‐value <.001 Fischer's Exact Test).

By comparing the answers to questions 5 and 6 of the questionnaire, we did not find statistically significant differences between the answers of those who agreed to the legalization of caesarean on maternal request (78.1%) and those of the obstetricians who ask patients about their preferred method of delivery (86.3%) (*P*‐value = 0.18 McNemar Test). However, we found statistically significant differences in the group who declared that they ask their patients how they preferred to deliver, and between those who agreed with legalized caesarean deliveries on maternal request (93.0%) and those who disagreed with the legalization (62.5%) (*P*‐value < .001 Fischer's Exact Test).

By comparing the answers to questions 7 and 9, we found statistically significant differences between the percentage of those who declared that they perform defensive CS (69.9%) and those who said they are influenced by the risk of malpractice when they choose the method of delivery for their patients (86.3%) (*P* < .001; McNemar Test). We also found statistically significant differences when comparing the percentage of those who declare they are influenced by the risk of accused of malpractice when choosing the method of delivery and those who declared that they perform defensive caesarean deliveries (100%); these percentages were similar to that of those who answered that they did not perform defensive CS (54.5%) (*P* < .001 Fischer's Exact Test).

## 4 DISCUSSION

Our study showed that defensive CS is performed in Romania and that it is widespread in the centers included in our study (69.9% of the respondents admitted under the protection of anonymity that they practice this type of intervention). The general opinion under the law is that in the gynaecology and obstetrics field, both young healthy women and the fetus are at risk. Defensive CS is usually chosen because it can diminish the possible substantial morbidity associated with vaginal delivery for the fetus and mother. Another contributing factor is the general risk avoidance attitude of society. The strength of our study is the high response rate of 100% for this survey. There was no refusal to participate. Factors that improved the response rate were that all respondents found the subject very interesting and appropriate because of the present status of obstetrics in Romania. Obstetricians who perform defensive CS do so to various extents which can exceed 50% of all CS deliveries performed. Indeed, 10 of 51 (19.6%) respondents who stated that they perform defensive CS considered that more than 50% of their deliveries are caesarean. Female obstetricians were more likely to agree with caesarean legalization on maternal request. Similar to our results, in one survey, 31% of the female obstetricians preferred performing CS.^10^ This is in contrast to a Dutch study which reported that only 1.4% of female obstetricians opted to perform caesarean deliveries.^11^

Of the 73 obstetricians in our study, 63 (86.3%) stated that the risk of being accused of malpractice influences the method of delivery they perform, which places intense pressure on the medical‐obstetrical professional body. In the literature, the relationship between malpractice claims payment and the use of CS is conflicting. Some studies indicated that many obstetricians view CS as a way of minimizing their exposure to litigation.^12^ Other analyses have found no such relation.^13^

Studies performed in the United States have indicated that 96% of neurosurgeons practice defensive medicine. Moreover, in Italy 94% of gastroenterologists and 85% of surgeons and anesthesiologists practice defensive medicine.^14^, ^15^ Romanian studies have revealed that the fear of a possible litigation is one of the iatrogenic factors that influence the frequency with which CS is performed, resulting in obstetricians resorting to a delivery method that involves a well‐standardized and understood technique and a higher degree of control.^16^ Studies have also shown that the use of certain manoeuvres and obstetrical instruments has decreased in Romania either because of the distress caused by possible legal issues or because of the emergence of newer generations of obstetricians who are not trained in the use of these manoeuvres or tools.^16^ It is important to emphasize that considering the current concerns regarding increasing caesarean delivery rates worldwide, the drive to reverse this trend is continued by training obstetricians for instrumental delivery, and the lack of experience and ability to select the appropriate instrument or manoeuvres can induce an increased rate of defensive CS.

Some authors have reviewed obstetrics‐related litigation as recorded by the Bucharest College of Physicians in 2011 to 2015.^16^ They collected information regarding 68 cases from 598 cases recorded for further investigations (approximately one‐tenth of the cases). Out of these, 49 were related to delivery or prenatal care. There were 22 cases in which patients expressed dissatisfaction because a CS was not performed at the time of delivery, even though caesarean delivery was not indicated. Medicolegal experts consider that in most cases of vaginal delivery, complications, such as brachial plexus paresthesia, neonatal hypoxia, meconium aspiration syndrome, episodes of neonatal epilepsy, cerebral palsy and fetal death, are considered to be preventable by CS delivery.^9^, ^16^ Additionally, obstetricians in Romania experience considerable work‐related stress, because medical cases and events are extensively discussed and popularized in the media, with obstetrics and gynaecology being one of the most targeted specialties. Patient dissatisfaction generates complaints that are often resolved in courts with doctors objecting to allegations of malpractice but paying thousands of euros in damages. Therefore, medical procedures are often less focused on helping the patient and are more focused on being defensive because the physicians are forced to work in a hostile social climate with increasingly higher pressures applied by the public.^9^, ^17^

Meanwhile, 60 of the 73 interviewed obstetricians (82.2%) considered it normal for the patient to express their preference on the type of delivery and 57/73 (78.1%) agreed with the legalization of CS on maternal request. Therefore, more than half of the respondents were in support of women having the right to choose CS as the mode of delivery. Accordingly, 63 out of the 73 obstetricians (86.3%) declared that they ask their patients about their preferred method of delivery, which suggests that they are willing to consider the wishes of the patient. Furthermore, they stated that obstetricians should inform their patients of this right. These data confirm that a large number of potential doctor‐patient litigations originate in the patient's desire and/or inclination towards a certain type of parturition (usually by caesarean delivery, which is considered safer, faster, and generates less discomfort) and the lack of some legislation that protects obstetricians when performing CS delivery as an alternative because of maternal request. In situations such as these, physicians attempt to produce medical indications that justify caesarean section under the current legal framework, which is similar to the system followed when performing a defensive CS. The attitude of society, patients, media, and the courts reflect a global intolerance to risk. One study emphasized the safe image of caesarean delivery in comparison to vaginal delivery and its possible morbidity.^18^ In our study, the consent or refusal of the patient's request for elective CS was not influenced by the different tertiary units. We must remember that objective obstetrical thinking could sometimes be dangerous and a more individualized approach is more suitable for managing cases involving defensive caesarean delivery and CS on maternal request.^19^

### 4.1 Limitations of study

This study has some limitations. First, the only settings were only in tertiary hospitals, and the questionnaire was not sent to other city hospitals in the country. However, because the demographic profile of respondents reflected that of Romanian obstetricians, the risks of bias were minimal. Second, the sample size was small. Since we tried to keep the questionnaire short we could not increase the validity of the study by including other questions about the topic. Unconscious defensive CS (in the context of defensive medicine) has not been reported by doctors, but it is also widely practiced.^20^ Further research regarding the cost of defensive caesarean delivery is also necessary.

## 5 CONCLUSIONS

Beyond medical indications for CS, defensive caesarean delivery is widespread in clinical practice in Romania, with 69.9% of respondents declaring that they perform defensive CS and 86.3% of obstetricians indicating that the risk of being accused of malpractice influences their performed method of delivery. Additionally, resident doctors in the study (the group of doctors with 0‐5 years of experience in obstetrics) have already acquired the habit of practicing defensive CS. Finally, most of the obstetricians who were interviewed (82.2%) believed that the patient has the right to choose the method of delivery. This prompts us to consider whether the introduction of CS upon maternal request would produce a substantial decrease in the practice of defensive caesarean delivery and in the number and severity of litigations. This might significantly reduce the pressure exerted on obstetricians, and it would imply that the quality and ethics regarding medical care would increase. Therefore, it is worth pondering whether the introduction of caesarean delivery upon maternal request in medical practice is the logical solution for a truly modern obstetrics.

## ETHICAL APPROVAL

No ethical approval required for this study.

## CONFLICT OF INTEREST

None declared.

## AUTHOR CONTRIBUTIONS

C.A.I. and M.D. contributed equally to this work by collecting and analysing the sample material and drafting the manuscript. M.B. and D.N. carried out textual analysis. E.P. performed statistical analysis. G.O.F. collected the sample material. L.P. also performed textual analysis. All authors revised the draft and read and approved the final submitted manuscript.
